# Religion and the Unmaking of Prejudice toward Muslims: Evidence from a Large National Sample

**DOI:** 10.1371/journal.pone.0150209

**Published:** 2016-03-09

**Authors:** John H. Shaver, Geoffrey Troughton, Chris G. Sibley, Joseph A. Bulbulia

**Affiliations:** 1 Religious Studies Programme, Victoria University of Wellington, Wellington, New Zealand; 2 School of Psychology, University of Auckland, Auckland, New Zealand; University of Amsterdam, NETHERLANDS

## Abstract

In the West, anti-Muslim sentiments are widespread. It has been theorized that inter-religious tensions fuel anti-Muslim prejudice, yet previous attempts to isolate sectarian motives have been inconclusive. Factors contributing to ambiguous results are: (1) failures to assess and adjust for multi-level denomination effects; (2) inattention to demographic covariates; (3) inadequate methods for comparing anti-Muslim prejudice relative to other minority group prejudices; and (4) *ad hoc* theories for the mechanisms that underpin prejudice and tolerance. Here we investigate anti-Muslim prejudice using a large national sample of non-Muslim New Zealanders (*N* = 13,955) who responded to the 2013 New Zealand Attitudes and Values Study. We address previous shortcomings by: (1) building Bayesian multivariate, multi-level regression models with denominations modeled as random effects; (2) including high-resolution demographic information that adjusts for factors known to influence prejudice; (3) simultaneously evaluating the relative strength of anti-Muslim prejudice by comparing it to anti-Arab prejudice and anti-immigrant prejudice within the same statistical model; and (4) testing predictions derived from the Evolutionary Lag Theory of religious prejudice and tolerance. This theory predicts that in countries such as New Zealand, with historically low levels of conflict, religion will tend to increase tolerance generally, and extend to minority religious groups. Results show that anti-Muslim and anti-Arab sentiments are confounded, widespread, and substantially higher than anti-immigrant sentiments. In support of the theory, the intensity of religious commitments was associated with a general increase in tolerance toward minority groups, including a poorly tolerated religious minority group: Muslims. Results clarify religion’s power to enhance tolerance in peaceful societies that are nevertheless afflicted by prejudice.

"…there is something about religion that makes for prejudice, and something about it that unmakes prejudice”—Gordon Allport [1]

## Introduction

Explaining anti-Muslim prejudice is both theoretically and practically important. Anti-Muslim sentiments are pervasive in Europe [2,3], the United States [4,5], Australia [6,7] and New Zealand [8]. Manifestations of this prejudice range from active avoidance to murder [3]. Explanations for anti-Muslim attitudes in Western countries have been the subject of longstanding and bitter debates. The present study evaluates theories that argue that prejudice and acceptance have a basis in inter-religious conflict. Our question is: *do the religious commitments of non-Muslims promote prejudice or acceptance*? As detailed below, existing studies offer ambiguous answers.

## Religion and Prejudice

### Individual Variation

Psychological studies investigating the relationship between religion and prejudice have produced inconclusive results. The first studies to address religion and prejudice conceptualized both religious affiliation and belief in God as binary variables [9,10]. Such studies–which we call *dichotomizing studies*–frequently observe a positive association between religious affiliation (yes/no), church membership and/or attendance (yes/no), or assenting to a belief in a God (yes/no) and generalized prejudice toward minority groups [11]. A meta-analysis of dichotomizing studies conducted between 1940 and 1990 found that in thirty-seven out of forty-seven studies, religiously affiliated people were more prejudiced than were non-religiously affiliated people (forty-four of these studies were conducted in the United States, two in the Netherlands, and one in London). By contrast, only two studies in this meta-analysis observed a negative association [9,12]. Notably, church attendance, also measured by a “yes/no” indicator of attendance, was associated with increased prejudice. A recent transnational study conducted across eleven European countries supports this inference: Europeans who report religious affiliation (yes/no) and attend church were found to exhibit greater prejudice toward minorities [13].

A limitation of dichotomizing-variable measures, however, is a loss of precision when assessing religious commitment. Some researchers who have measured church attendance as a continuous variable have observed a curvilinear relationship between frequency of attendance and prejudice [14]. In a meta-analysis of 25 studies (*N* > 21, 936; twenty-four studies conducted with American participants, one with British, Welsh and Dutch participants) Gorsuch and Aleshire [15] found that those who attended church at intermediate rates were more prejudiced than those who attended church frequently and those who never attended.

It has been theorized that the link between church attendance and religious prejudice arises from differences in the way people value religion, that is, from different “*Religious Orientations*” [15]. Though different typologies for religious orientations have been proposed and defended, there appears to be a stable link between how individuals value their religion and how individuals value other people (i.e., moral judgments) [16]. Studies that have investigated religious orientations and prejudice find that people who are more flexible in their orientation to their faith (i.e., are Quest oriented), or value their faith for intrinsic reasons (i.e., are Intrinsically oriented), tend to exhibit less prejudice toward minority groups; by contrast, those who are motivated toward religion for personal gain (i.e., are Extrinsically oriented) or have rigid beliefs (i.e., have a Fundamentalist orientation) tend to exhibit greater prejudice [1,9,10,17,18]. Religious Orientations theorists propose that the overall negative relationship between religion and prejudice is an artifact of the greater frequency of extrinsic religious orientations, yet some types of religious orientations attenuate prejudice [1,9,11].

Religious Orientations theories improve on dichotomizing studies because they attempt to account for within group heterogeneity. Despite attempts in the Religious Orientations literatures to identify religion's role in the making and unmaking of prejudice, however, empirical results have been ambiguous. The increased tolerance associated with some religious orientations does not appear general; a recent meta-analysis (*N* = 5,861; twenty-five studies; no indication of countries sampled) has shown that some orientations are associated with greater tolerance toward some groups but increased prejudice toward others [10]. For example, Canadian Christians report that prejudice against some out-groups is proscribed (e.g., racism), while prejudice against others is not forbidden and may in some cases even be encouraged (e.g., against homosexuals) [10]. More fundamental, Religious Orientations theories do not explain why intrinsic motivations are systematically associated with greater support for out-group violence—the opposite of tolerance—in some regions but not others [19,20]. For instance, Palestinians and Israelis who are committed to their respective religions are more likely to endorse out-group violence [20]. Finally, the theoretical account of why intrinsic religion should function to reduce prejudice is unconvincing because religious traditions, authorities, and texts do not invariably promote tolerance. Put simply, then, Religious Orientations theory has trouble accounting for variation in prejudice, both within and between cultures. To clarify the mechanisms of anti-Muslim prejudice, we argue that both fundamental theoretical and statistical shortcomings need to be addressed.

### Cultural variation

Historical studies investigating the relationship between religion and prejudice have also produced ambiguous results. One of the most widely accepted theories—which we call the *Clash of Civilizations Model*—observes that conflict between Muslims and Christians has deep historical antecedents and predicts that such conflict will worsen in years to come [21,22]. The theory posits that sectarian tensions are self-perpetuating: religious conflict elaborates more religious conflict, a cycle that escalates conflict over time.

Although there have been centuries of Christian-Muslim conflict, the *Clash of Civilizations* model fails to account for regional variation in intergroup-relations; Muslim tensions among traditionally Christian nations are not regionally uniform [23,24]. Cross-cultural data drawn from the European Values Survey (EVS) reveals that across forty-seven countries, anti-Muslim prejudice ranged widely. For instance, in 2008 in Iceland, 7.5% of people reported that they would not like to have Muslims as neighbors, while in Lithuania this number rose to 46.7% [25]. Moreover, EVS data show that prejudice toward Muslims was substantially greater than toward immigrants in most, but not all, countries of Western and Eastern Europe, in both 1999 (higher in 22/29 countries) [26] and again in 2008 (higher in 34/47 countries) [25]. In some countries there was no difference between immigrant and Muslim prejudice, while in others, generalized immigrant prejudice was higher than Muslim prejudice.

In terms of religion and anti-Muslim prejudice, a study using the 1999 EVS data (*N* = 41,000 participants) found no evidence for a reliable association between anti-Muslim attitudes and either strength of religious belief or religious attendance or self-rated importance of God [26]. The authors conclude: "results give us little reason to believe that the religious component in itself is a prominent factor in the generation of anti-Muslim prejudice" [26]. By contrast, a separate study using 2008 EVS data collected among 44 countries (*N* = 54,740 participants) found that several dichotomous measures such as belief in God, and belief in a "life force," were associated with lower prejudice toward Muslims, while church attendance was unrelated to anti-Muslim prejudice [25]. Meanwhile, using data drawn from the 2008 British Social Attitudes Survey (*N* = 2,250), Clements [27] did not find religious affiliation to be reliably associated with anti-Muslim sentiment, while the salience of religion in a person’s life was associated with greater acceptance of Muslims.

While the *Clash of Civilizations Model* usually points to historical path dependencies, there is also evidence that the association between religion and inter-group conflict varies across time and space [19,28,29]. We contend that variation in religion’s association with conflict is systematic, and can be explained with reference to cultural evolutionary dynamics. Our study uses evolutionary theory to clarify the mechanisms by which religion affects anti-Muslim prejudice in affluent and peaceful Western societies where such prejudice is commonplace [30].

## The Evolutionary Lag Theory of Prejudice and Tolerance

Evolutionary approaches to religion are comprehensive and include historical/regional and psychological factors within a unified conceptual framework [31,32]. Empirical applications of evolutionary theory have been highly successful in explaining geo-political variation in religion [29,33], regional associations between religion and war [28,34,35], and in clarifying the proximate psychological mechanisms motivating these processes [36,37].

Evolutionary theories approach religious systems as evolved mechanisms for coordinated social action [38,39]. Evolutionary theorists observe that human social groups are highly cooperative, but that collective resources are vulnerable to exploitation by self-interested individuals [40]. Many scholars now believe that religious cultures co-evolved to support pro-sociality and/or suppress anti-social behavior [38,39,41,42]. We argue that historically, some religious systems may have been more effective at limiting self-interest and promoting within group cooperation and out-group aggression; in combination with political factors (e.g., aggressive colonization) and technological factors (e.g., military superiority), such characteristics helped a few religious traditions achieve global dominance. Though the jury is still out on cultural evolutionary theories of religion, both scholars of religion and evolutionary theorists agree that religions are strategically flexible systems that vary in response to local natural and social ecologies [39,43–46]. Despite attention to the role of religion in political violence, few models of religion and inter-group prejudice are explicitly informed by evolutionary theory.

The strategic flexibility of religions predicts regional variation in aggression and tolerance in response to local environments and histories [35,47]. Religious doctrines, in particular, are flexible [48]. The religious texts of the world’s major religions overwhelmingly promote peace [49], but they also legitimate violence and conflict [50]. In environments with high levels of conflict due to threats to collective resources, religious leaders often emphasize the violent sides of religions and direct hostility toward out-group members [51,52]. In these environments, highly religious individuals internalize these aspects of religious teachings and endorse out-group hostility and prejudices. Conversely, in peaceful environments, where religious teachings focus on pro-sociality and tolerance, high levels of internalization will be associated with greater tolerance. For example, Ginges et al. [20] found that Palestinians and Israelis who report higher levels of religious commitment were more likely to endorse violence against the other group. In other words, in certain regions where there is violent conflict, religious commitments appear to exacerbate out-group hostility. Conversely, in regions with little violent conflict, religious commitments appear to attenuate out-group distrust. For example, American Christians report that they trust devout Muslims more than less religious Muslims [53].

To explain variation in the association between religion, peace, and conflict, we propose an *Evolutionary Lag Theory* according to which religious cultures exhibit strategic features that are sensitive to local and historical inter-group peace and conflict: in geo-political settings where there is an ongoing history of intergroup competition for resources, the Evolutionary Lag Theory predicts that religious commitments will tend to increase [54] and manifest as negative attitudes toward out-groups. By contrast, the Evolutionary Lag Theory predicts that in environments with a history of low levels of competition and peaceful contact between religious groups, identification with a religion will tend to foster higher levels of tolerance toward other religious groups. Here we investigate the predictions of the Evolutionary Lag Theory using data collected in New Zealand, a peaceable country with little history of religious conflict.

## Research Setting: Muslims in New Zealand Society

For a host of reasons, New Zealand is an ideal setting in which to investigate mechanisms that drive prejudice in the absence of violent conflict.

First, New Zealand’s cultural diversity exceeds that of Australia, the United Kingdom, France, Germany, the Netherlands, and Scandinavia [55]. At the most recent national census in 2013, New Zealand had a population of 4,242,048, with 668,724 (16%) people reporting Maori, or indigenous, ancestry [56]. According to the New Zealand census, the first members of the Islamic community arrived by at least 1874 as a small group of male migrant laborers, probably from China, but all eventually returned to their native countries [57]. Then, beginning in the early part of the 20th century, Muslims began to permanently settle in New Zealand, with this first wave of Muslim immigrants comprised of a small group of Gujarati men who ran small shops south of Auckland [57]. The New Zealand Muslim community remained modest in size for the first half of the 20th century and numbered only 205 at the time of the 1951 census. More recently, however, the Muslim population has increased dramatically from 5,772 in 1991, to 46,149 in 2013 [56]. The current New Zealand Muslim community is of diverse origins—of those that affiliated with Islam in 2013, 26.9% were born in Asia, 25.7% in New Zealand, 23.3% in the Middle East or Africa, and 21% in the Pacific Islands [56].

Second, New Zealand is one of the world’s most socially progressive and tolerant societies [58,59], and has a long tradition of religious tolerance and accommodation of religious diversity. Principles of religious equality were affirmed as early as 1840 at the signing of the Treaty of Waitangi, which paved the way for British annexation and colonization [60]. Crucially, the colonial New Zealand state never adopted an official state religion, while principles of religious equality and the promotion of social harmony led to the formation of a secular state primary education system beginning in 1877 [61,62]. Antipathy to non-white migration was evident at times. Sectarian convulsions, notably between Catholic and Protestant Christians, also occasionally erupted, particularly around the period of WWI. Commentators note, however, that the extent and intensity of sectarian hostility was usually less pronounced in New Zealand than in other similar nations such as nearby Australia [63]. In a small, religiously diverse colony where pragmatism and egalitarian values were highly esteemed, religion-based conflict returned few benefits [62]. The ethno-cultural and denominational diversity in the nation may also have contributed to the relative harmony that existed between religious traditions.

Third, interactions between Muslims and New Zealand’s other ethnic groups–including the ethnic European majority–are generally peaceful. However, non-violent conflicts have been reported [30]. For example, in recent years members of Islamic communities have been subjected to mild forms of harassment, vandalism, negative media portrayal, and/or have faced difficulties in the labor market [30]. However, overall, violent conflict between Muslims and other groups is, at present, minimal. Thus, anti-Muslim attitudes are unlikely to be grounded in negative interpersonal experiences.

Fourth, when violent harassment of Muslims has occurred, there have been inter-faith responses. For example, in 1998 arsonists burned a mosque in Hamilton, six months after it opened. In November 2005, shortly after the London bombings, two white men vandalized a mosque in Auckland. Following the 1998 mosque burning, however, members of the Christian and Jewish communities publicly spoke out against the attack. Similarly, after the Auckland mosque was vandalized, members of local non-Muslim religious communities organized an inter-faith rally in support of Muslims. More generally, there are growing examples of interfaith institutions in New Zealand during the past several decades, and such institutions are now present in most major cities. Some of these institutions attempt to link Muslims, Christians, and Jews by emphasizing their shared identity as “Abrahamic” religions. For example, in Dunedin, an Abrahamic Interfaith Group was explicitly formed as a response to the events of September 11, 2001. In 2015 the former Wellington Council for Christians and Jews was reconstituted as the Wellington Abrahamic Council in an attempt to foster greater harmony between people from different religious and ethnic backgrounds. Put simply, a wealth of anecdotal evidence suggests that religion may be a driver of peaceable relations with New Zealand’s Muslims.

Fifth, New Zealand society is largely secular; as of 2013, close to half of the population (1,635,345/41%) reported that they had no religion. The large numbers of religiously unaffiliated people in New Zealand allows for comparisons between religious and non-religious people who are similar in respects to education, political orientation, socio-economic status, and other demographic factors that previous research has suggested are associated with tolerance and prejudice.

Finally, New Zealand is home to The New Zealand Attitudes and Values Study (NZAVS), a 20-year longitudinal study of attitudes and values in a large and diverse sample of New Zealanders (www.nzvalues.org). The study includes rich and reliable measures of demographic and political variables known to affect prejudice and tolerance, measures of religious identification, church/temple attendance, indicators of denominational affiliation, as well as fine-grained regional information about relative deprivation [64]. Here we investigate anti-Muslim prejudice using a non-Muslim, non-immigrant sub-sample of New Zealanders who responded to the 2013 New Zealand Attitudes and Values Study (*N* = 13,955).

## The Study and Its Predictions

We address the shortcomings of previous research in a variety of ways. First, our multi-level statistical models adjust for dependencies arising from religious denominations. Importantly, we do not test a theory of denominational difference. Indeed, the Evolutionary Lag Theory is skeptical that general denominational differences drive religious prejudice and acceptance; rather, historical and regional path dependencies give rise to strategic settings to which religious institutions both contribute and respond. Nevertheless, denominational differences might arise, and tend to affect people who identify with a denomination in similar ways, giving rise to dependencies in judgments. Even small dependencies are known to invalidate the statistical assumptions of regression, leading to anti-conservative estimates of standard errors in regression coefficients (i.e. artificially reducing the standard errors) [65, 66]. Second, we obtain a comparative understanding of anti-Muslim prejudice by evaluating the relative differences of anti-Muslim, anti-Arab and anti-immigrant sentiment as multivariate outcomes with the same statistical models that evaluate religion’s association with prejudice. This enables us to effectively leverage information about potentially different types of minority-group prejudice within the same statistical model, and to assess the correlations between these different types of prejudice. Finally, the theoretical model we test is grounded in evolutionary theory, and informed by a strategic assessment of religion in cultural-historical context.

New Zealand’s history of religion-supported tolerance and low levels of inter-group competition lead to the prediction that levels of religious commitment will be associated with correspondingly increasing levels of acceptance. Additionally, New Zealand’s religious communities have longstanding historical commitments to social equality [67,68]. Hence, we predicted that religious identification in New Zealand would also be associated with less prejudice toward Arabs and immigrants. In other words, we expected that people who were religiously identified would express lower levels of prejudice toward all three target groups. We assessed religious commitment in two ways: levels of subjective identification with a religion and rates of church attendance.

## Method

### Sampling Procedure

The New Zealand Attitudes and Values Study is reviewed every three years by the University of Auckland Human Participants Ethics Committee. Our most recent ethics approval statement is as follows: The New Zealand Attitudes and Values Study was approved by The University of Auckland Human Participants Ethics Committee on 03-June-2015 until 03-June-2018. Reference Number: 014889. Our previous ethics approval statement for the 2009–2015 period is: The New Zealand Attitudes and Values Study was approved by The University of Auckland Human Participants Ethics Committee on 09-September-2009 until 09-September-2012, and renewed on 17-February-2012 until 09-September-2015. Reference Number: 6171. All participants granted informed written consent and The University of Auckland Human Participants Ethics Committee approved all procedures.

The New Zealand Attitudes and Values Study (NZAVS) is an annual, longitudinal national probability sample of registered New Zealand voters, which was started in 2009. We analyzed data from participants who completed the Time 5 wave of the NZAVS. The Time 5 (2013) wave of the NZAVS contained responses from 18,264 participants (10,502 retained from one or more previous waves, 7,581 new additions from booster sampling, and 181 unmatched participants or unsolicited opt-ins). The sample retained 3,934 participants from the initial Time 1 (2009) NZAVS of 6,518 participants (a retention rate of 60.4% over four years), and 9,844 participants from the full Time 4 (2012) sample (a retention rate of 80.8% from the previous year). The most recent wave of NZAVS responses that have been entered is from 2013 (Time 5). For this wave, participants were mailed a copy of the questionnaire, with a second postal follow-up two months later. Participants who provided an email address were also emailed and invited to complete an online version if they preferred. We offered a prize draw for participation, non-respondents were emailed and phoned multiple times, and all participants were mailed a Season’s Greetings card from the NZAVS research team and informed that they had been automatically entered into a bonus seasonal grocery voucher prize draw. We also mailed our yearly pamphlet summarizing key research findings published during the current wave of the study.

### Participants

The Time 5 (2013) wave of the NZAVS included 18,264 respondents. Of these participants, 30 were removed due to missing or incomplete information about their age, resulting in a sample of 18,234. Of these participants, 40 self-identified as Muslim, 41 as Middle Eastern and 3,607 as immigrants (i.e. not born in New Zealand). Moreover, 420 did not indicate a country of birth. Because we were interested in out-group determinants of prejudice toward Muslims, Arabs, and immigrants, only New Zealand-born non-Muslim participants were included in the analysis. Of the 41 people who identified as Middle Eastern, 13 stated both that were born in New Zealand and that they identified with a faith other than Islam (including no faith). Though not all people of Middle Eastern ancestry identify as Arab (for example Iranians may identify as Persian, and Israelis may identify as Jewish) we excluded those participants who identified as Middle Eastern to avoid unintentionally modeling attitudes among people who identify as Arab. This resulted in a sample of *N* = 13,955.

### Correlation of Prejudice Types

We argue that the extent to which anti-Muslim prejudice differs from other forms of prejudice remains unclear because the relationship between anti-Muslim prejudice and other forms of prejudice has not been simultaneously investigated, and thus, a comparative understanding of anti-Muslim prejudice is lacking. Although some studies have examined whether there are differences in the means of anti-Muslim prejudice and anti-immigrant prejudice [25,26], information is more efficiently pooled when types of prejudice are simultaneously modeled as multivariate outcomes. Simultaneously modeling associations between demographic and religious covariates (predictors) on anti-Muslim prejudice and appropriate comparison groups (multivariate outcomes) affords a comparative assessment of the mechanisms that influence prejudice because it adjusts for within-individual co-variation in responses.

### Measures of Prejudice

**Fig 1:**
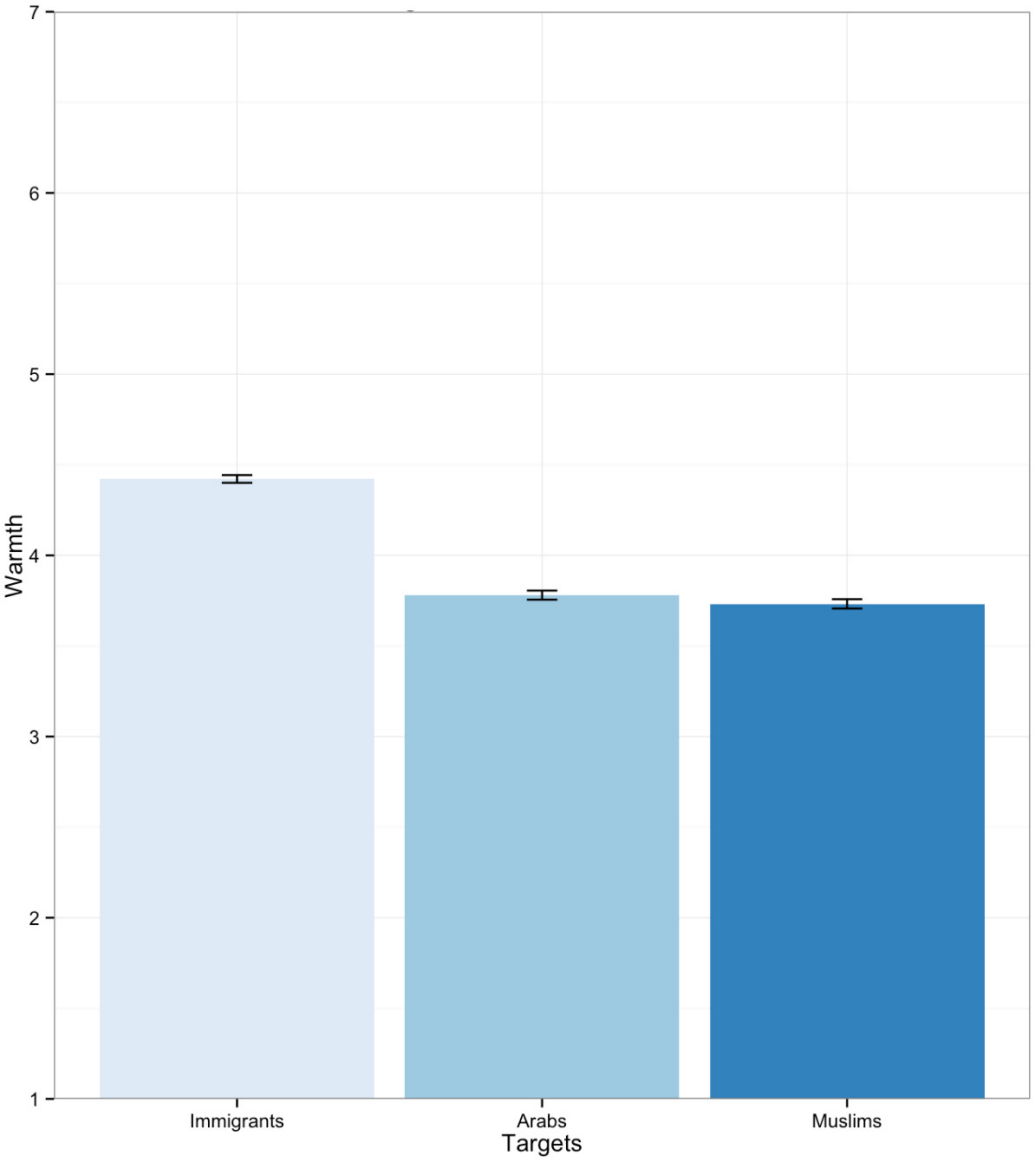
Warmth towards Immigrants, Arabs and Muslims.

Affective thermometer ratings were used to assess prejudice toward Muslims, Arabs and immigrants by asking participants to indicate the “warmth” they feel toward these groups on a scale ranging from 1 (least warm) to 7 (most warm), with 4 (neutral) as the midpoint. Affective thermometer ratings are widely used measures of positive/negative attitudes toward social groups both in New Zealand [69] and abroad [70]. Mean levels of warmth toward the three focal target groups is presented in Fig 1. A summary of all variables used in analyses is presented in Tables 1 and 2.

**Table 1:** Interval/Ordinal Variables Used in Analyses.

Variable	Mean	Standard Deviation	Range	Number Missing	Percentage of Data Missing
**Warmth toward Immigrants**	4.42	1.27	1 to 7	238	0.017
**Warmth toward Arabs**	3.78	1.50	1 to 7	267	0.019
**Warmth toward Muslims**	3.73	1.56	1 to 7	248	0.018
**Age**	47.4	14.10	15 to 94	0	0.001
**Education**	.263	1.10	- 1 to 2	1,484	0.106
**Political Conservatism**	3.66	1.27	1 to 7	836	0.060
**Deprivation**	4.91	2.80	1 to1 0	238	0.017
**Religious ID**	1.79	2.59	0 to 7	219	0.016
**Church Attendance**	0.81	4.81	0 to 400	162	0.012

**Table 2:** Dichotomous Variables Used in Analyses.

Variable	Proportion	Number Missing	Percentage of Data Missing
**Employed**	0.765	115	0.008
**Male**	0.366	1	0.000
**Parent**	0.732	0	0.000
**European Descent**	0.912	0	0.000
**In a Relationship**	0.704	0	0.000
**Urban**	0.649	121	0.009

### Theoretical Variables

#### Religious Identification

To assess religious identification, we asked people: “Do you identify with a religion and/or spiritual group?” (yes or no). For those who identified with a religion, we asked participants to rate on a scale from 1–7 “how important is your religion to how you see yourself?” Those individuals who indicated that they did not belong to a religion were coded as a 0 (*N* = 8,545) on this scale (*M* = 1.79; *SD* = 2.60).

#### Church Attendance

Church attendance was assessed by asking participants how many times they attended church or a house of worship in the past month (*M* = 0.81, *SD* = 4.83) [71]. Those who did not report a religious affiliation were assigned a response of zero. Because church attendance rates varied considerably we obtained a linear transformation of church attendance using the natural logarithm to yield a log scaled church attendance indicator.

We assessed a person’s connection with their religion using both Religious Identification and Church Attendance because we expect these constructs to measure different aspects of religious experience. People may highly identify with a group even though they rarely attend a house of worship (e.g., people who have health problems, or feel disaffected with the local church), and some may attend church often but do not highly identify with an organized religion (e.g., if one attends at the request of a spouse, or those who attend for business connections). The differences across these two dimensions may manifest in various ways to influence prejudice and tolerance. For example, people may come to hold negative attitudes toward an out-group because they highly identify with the in-group, or because they spend substantial time with in-group members.

Multicollinearity arises when predictors are highly correlated, and results in inflated standard errors for regression coefficients (i.e. excessive conservatism). Variance Inflation Factors (VIFS) were calculated for the full univariate outcome models using maximum likelihood estimation. All VIFS were below 1.5, indicating that the variance inflation was not a problem. In other words, though religious identification and church attendance were positively correlated (τ = .619), diagnostics suggested that the inclusion of both in the same predictive model is warranted. Further data exploration revealed differences between these measures: of those who score at the top of the religious identification scale (7), 39% reported attending church/temple less than once a week and 21% report not attending church at all.

### Demographic Indicators

Studies have shown that several individual level demographic variables may be associated with prejudice. For example, people with more education often exhibit lower prejudice [72,73], as do women [74], people of higher socio-economic status [75,76], the politically liberal [77], residents of urban areas [13,78], younger people [79], and employed people [80,81]. We therefore included these demographic variables in all models. Moreover, because previous NZAVS research has revealed that European ethnic affiliation (yes/no), relationship status (yes/no) and parental status (yes/no) are each associated with many psychological and social outcomes [82–84], we also included these variables in all analyses.

#### Age

The mean age of the sample was 47.42 (SD = 14.09).

#### Education

Education was coded as either some high school “-1” (n = 4,996), high school diploma/certificate, “0” (*n* = 3,571), undergraduate degree “1” (*n* = 4,295), or *"2"* post-graduate education (*n* = 2,993).

#### Employment

Employment status was assessed by asking participants if they were currently working, “yes” was coded as “1” (*n* = 10,586) and “no” was coded as “0” (*n* = 3,254).

#### Gender

The sample included 5,074 males (coded as 1) and 8,880 females (coded as 0).

#### Parental Status

We assessed parental status by asking participants to indicate their number of children. Participants were coded as “0” if they reported that they do not have children (*n* = 3,735) and “1” if they reported that they do (*n* = 10,220).

#### Political Liberalism/Conservatism

Political orientation was assessed using a single-item that asked participants to report their political orientation on a 1 (Liberal) to 7 (Conservative) scale (*M* = 3.66, *SD* = 1.27).

#### European Ethnic Affiliation

All participants in the sample reported that New Zealand was their birthplace. However, many New Zealanders identify with the ethnicities of ancestors who migrated to New Zealand. We also assessed ethic origin, and 12,739 participants indicated that they were of European descent (coded as 1), while 1,216 indicated non-European ancestry (coded as 0).

#### Relationship Status

Participants were asked if they were in a relationship, “yes” was coded as “1” (*n* = 9,821) and no was coded as “0” (*n* = 4,134).

#### Deprivation/Socio-Economic Status

We measured the socio-economic status of participants’ immediate (small area) neighborhood using the 2013 New Zealand Deprivation Index [85,86]. New Zealand is unusual in having rich census information about each area unit/neighborhood of the country that is made available for research purposes. The smallest of these area units are meshblocks. The NZAVS includes the meshblock code for each participant.

The geographic size of these meshblock units differs depending on population density. Each unit tends to cover a region containing a median of roughly 81 residents (*M* = 95.95, *SD* = 73.49, range = 0–1899). In 2013 there were a total of 44,211 meshblocks for which data was available.

The New Zealand census [56] defines a meshblock as “a defined geographic area, varying in size from part of a city block to large areas of rural land. Each meshblock abuts against another to form a network covering all of New Zealand including coasts and inlets, and extending out to the two hundred mile economic zone. Meshblocks are added together to ‘build up’ larger geographic areas such as area units and urban areas.”

The New Zealand Deprivation Index [85,86] uses aggregate census information about the residents of each meshblock to assign a decile-rank index from 1 (most affluent) to 10 (most impoverished) to each meshblock unit. Because it is a decile-ranked index, the 10% of meshblocks that are most affluent are given a score of 1, the next 10% a score of 2, and so on. The index is based on a Principal Components Analysis of the following nine variables (in weighted order): proportion of adults who received a means-tested benefit, household income, proportion not owning own home, proportion single-parent families, proportion unemployed, proportion lacking qualifications, proportion household crowding, proportion no telephone access, and proportion no car access.

The New Zealand Deprivation Index thus reflects the average level of deprivation for small neighborhood-type units (or small community areas of about 80–90 people each) across the entire country. The index is a well-validated index of the level of deprivation of small area units, and has been widely used in health and social policy research examining numerous health outcomes, including mortality, rates of hospitalization, smoking, cot death, and access to health care, to name just a few examples [87–90]. The index is also widely used in service planning by government and local council, and is a key indicator used to identify high needs areas and allocate resources such as health funding [91,92]. Our sample had a mean deprivation index of 4.91 (*SD* = 2.80).

#### Urban/Rural

People were coded as either residing in an urban “1” (*n* = 8,971) or rural “0” (*n* = 4,863) area based on New Zealand census data.

### Statistical Analyses

#### Imputation

Missing data frequencies were relatively low across responses to most variables, with missingness typically observed at less than 2% (Tables 1 and 2). An exception was in responses to education, where missing responses were observed for 10.6% of the participants. We adopted two strategies for handling missing data. First, we modeled associations using pairwise deletion of data rows where missingness was observed. Well-known problems with pairwise deletion include reductions in efficiency from lost information and biased estimates [93]. Second, we modeled associations using multiple imputation. Multiple imputation of missing data preserves information and attenuates the effects of response biases in conditions where the causes of missingness may be predicted from other observed variables [93]. For this reason we prefer multiple imputation, and report multiply-imputed datasets in the main text of this study, while pairwise deleted models are reported in the supplement. In this study, we found that that there were no practically important differences in statistical inference between the pairwise deleted and multiply imputed strategies. Though multiple imputation was our preferred option, we caution that it is not entirely satisfactory. Multiple imputation cannot adjust for biases arising from factors that are not included in the imputation model [94].

Missing data were multiply imputed using both the Mice package [95] and the Amelia package [96] in R [97]. We also ran identical models using pairwise deleted data. In the main text we present the results of a model run on data imputed with the Mice package, however all three methods for handling missing data were similar (e.g., S1 Fig, and S1–S4 Tables, and S1 File and S2 File). For data imputation, nominal responses (factors): European Ethnicity, Male Gender, Employment Status, Parent Status, Partner Status, and Urban Location. “Denominations” (a random effect) and "Warmth To Arabs", "Warmth to Immigrants,” “Warmth to Muslims” (response variables) were not imputed but rather estimated during MCMC, (which is allowed by MCMCglmm package R version 3.2.1 [98]). The remaining missing variables were assumed to be continuous real numbers. Following Amelia package recommendations, where low frequencies of missing responses are observed, we imputed five missing datasets. Because the models were conditionally independent, multivariate multi-level regression models were performed separately on each dataset, and the MCMC chains were subsequently combined to obtain aggregate estimates. Models using the pairwise deletion strategy produced coefficients and intervals that only slightly varied from multiple imputation results, but again theoretical inference was not affected.

#### Denominations

Previous studies have failed to adjust for multi-level dependencies arising from participants’ affiliation with religious traditions [99]. Regression and ANOVA assume the statistical independence of errors, however this assumption may be violated when belonging to a religious tradition causes members to respond in predictable ways. It is widely regarded that conditional independence of errors is one of the most important assumptions of regression and ANOVA [100].

To adjust for dependencies owing to denominational clustering, we modeled denominational intercepts as random-effects. Intercept variances were centered at zero, and variance/co-variance coefficients were estimated for denominations. Participants were classified using the 2013 New Zealand census categories, which contains 93 categories. The category “no religion” was given to those who did not state a religious identification. In the current sample, 70 denominational categories were represented, 81 participants reported affiliations that could not be classified, 2 objected to answering, and there were 374 missing responses (e.g., S5 Table). Though many denominations were only sparsely represented in our sample, and all differed in size, in a mixed-effects model, group co/variance estimates are weighted by the number of observations in each group [101], with the group mean centered at zero, and only variance components (typically) estimated. It is important to note that though we adjusted for denominational influence to handle potential sources of multi-level dependency, we did not test a theory of any specific denominational difference as such (e.g. whether Quakers are more peaceable than Buddhists). We tested a model according to which, on average, religious commitment would predict tendencies toward tolerance, even after adjusting for denominational clustering.

#### Bayesian regression

We modeled attitudes toward minority groups using Bayesian multivariate mixed-effects regression. A key advantage of Bayesian regression is that it allows the pooling of information when estimating uncertainty for parameters of theoretical interest [101]. In Bayesian regression, there is little distinction between fixed and random effects [101,102], however the convention helps to identify model features.

Priors for the fixed components of the model were uninformative, with a mean of zero and variance of 10^8^. Following Hadfield [103] we used parameter expanded priors to adjust for the variance of denominations. Parameter expanded priors were centered at zero, and assumed a variance of 10^2^, which was only weakly informative on this dataset. Priors on residual variances were centered at zero, and assigned a normal inverse Wishart distribution that was not informative, meaning that the variance component estimates were informed by the data and not the priors. Though strong priors are preferable in many designs, the size and diversity of our sample warrants the use of non-informative priors.

To facilitate interpretation and MCMC mixing, religious identification, political conservatism and deprivation were centered and standardized. The model’s intercept terms are interpretable as the expected outcomes when predictors are set to zero, in this case: the expected level of warmth in the population of respondents who are average sample age (47.40 years old), high school educated, do not identify as European, not-religiously identified, non-church attending, female, of average political conservatism, without any partner, without any children, of average socio-economic status, and living in a rural setting.

The default number of MCMC cycles in MCMCglmm is 13,000, with a burnin of 3,000 cycles and a thin interval of 10 [102]. We ran models for 53,000 cycles with a burnin of 3,000 cycles and a thin interval of 10. Evidence from plots of the posterior distributions for all models indicated MCMC chains mixed well and there was no evidence of significant auto-correlation in the chains. Effective samples were all well over 1,000.

We follow those who urge that statistical inference is best conceived as a process of estimating unknown and in most cases unknowable population parameters as best one can, with a clear appreciation about the inevitable limitations that confront inferences based on inherently uncertain magnitudes and systems [103]. Bayesian regression produces results with transparent and intuitive probabilistic interpretations: the posterior distributions that are generated from MCMC are probabilistic distributions for modeled associations, which are conditional on the data, model, and priors.

## Results

**Fig 2:**
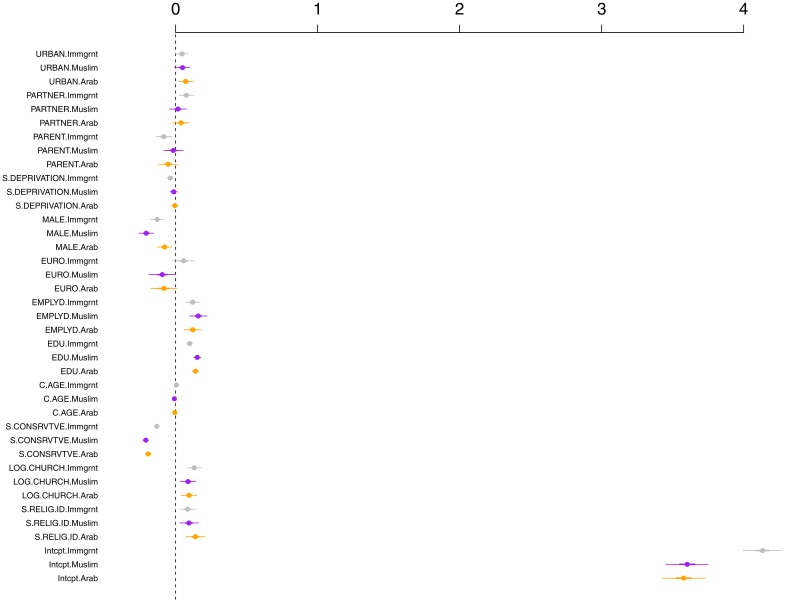
MCMC Solutions: Warmth to Arabs, Muslims, Immigrants.

The results of a multivariate regression model predicting warmth toward immigrants, Arabs and Muslims are presented in Table 3 and Fig 2. We first modeled prejudice using the intercept-only model with random denominational effects. We used the Deviance Information Criterion (*Δ* DIC) to assess the improvement of the full model compared to the intercept-only model [104]. Generally, a reduction of 10 or more on the DIC is regarded to be an improvement of model fit. The DIC score for the intercept only model including was *Δ* DIC = 122,909.1, and for the full model was *Δ* DIC = 121,360. In other words, the fit of the full theoretical model substantially improved the intercept-only model *(Δ* DIC = -1549). The estimates for the intercepts in the full theoretical model were as follows: warmth toward Muslims *b* = 3.602, [HPD interval from 3.459 to 3.753], Arabs *b* = 3.577 [HPD interval from 3.429 to 3.728], and Immigrants *b* = 4.133, [HPD interval from 4.003 to 4.273].

**Table 3:** Predictors of Tolerance toward Immigrants, Arabs and Muslims.

	Warmth toward Immigrants				Warmth toward Arabs				Warmth toward Muslims			
	Posterior Mean	95% Lower Bounds	95% Upper Bounds	pMCMC	Posterior Mean	95% Lower Bounds	95% Upper Bounds	pMCMC	Posterior Mean	95% Lower Bounds	95% Upper Bounds	pMCMC
**Intercept**	**4.133**	**4.003**	**4.273**	***	3.577	3.429	3.728	***	3.602	3.459	3.753	***
**Age (centered)**	.006	.004	.007	***	-.006	-.008	-.004	***	-.009	-.011	-.007	***
**Education**	.099	.080	.120	***	.140	.114	.162	***	.152	.127	.177	***
**Employed**	.120	.066	.168	***	.121	.060	.179	***	.159	.099	.219	***
**Gender**	-.130	-.177	-.087	***	-.079	-.129	-.026	***	-.207	-.260	-.156	***
**Parent**	-.084	-.137	-.027	***	-.053	-.118	.0134		-.016	-.081	.053	
**Political Conservatism (standardized)**	-.133	-.151	-.116	***	-.193	-.213	-.171	***	-.210	-.232	-.189	***
**European**	.055	-.022	.133		-.082	-.174	.005		-.094	-.188	-.002	*
**Partner**	.075	.024	.126	**	.038	-.017	.098		.016	-.044	.075	
**Deprivation (standardized)**	-.038	-.059	-.015	***	-.006	-.032	.018		-.014	-.041	.011	
**Urban**	.045	.000	.088	*	.070	.018	.123	**	.048	-.006	.102	
**Religious ID (standardized)**	.084	.027	.135	**	.140	.078	.205	***	.094	.030	.162	**
**Church Attendance (log)**	.131	.084	.179	***	.094	.040	.149	***	.088	.030	.140	**

* = pMCMC < 0.05

** = pMCMC < .01

*** = pMCMC < .001

### Demographic Indicators

#### Age

Each year of age was associated with more warmth toward immigrants (*b* = 0.006, HPD interval from 0.004 to 0.007, pMCMC < .001), but less warmth toward Arabs (*b* = -0.006, HPD interval from -0.008 to -0.004, pMCMC < .001) and Muslims (*b* = -0.009, HPD interval from -0.011 to -0.007, pMCMC < .001).

#### Education

Educated people were warmer toward immigrants (*b* = 0.099, HPD interval from 0.080 to 0.120, pMCMC < .001), Arabs (*b* = 0.140, HPD interval from 0.114 to 0.162, pMCMC < .001) and Muslims (*b* = 0.152, HPD interval from 0.127 to 0.177, pMCMC < .001). Education was associated with a larger increase in warmth for Arabs and Muslims than for immigrants.

#### Employment

Employment was associated with more warmth toward Muslims (*b* = 0.160, HPD interval from 0.099 to 0.219, pMCMC < .001), Arabs (*b* = 0.121, HPD interval from 0.060 to 0.179, pMCMC < .001) and immigrants (*b* = 0.120, HPD interval from 0.066 to 0.168, pMCMC < .001).

#### Gender

Men reported less warmth toward immigrants (*b* = -0.130, HPD interval from -0.177 to -0.087, pMCMC < .001), Arabs (*b* = -0.079, HPD interval from -0.129 to -0.026, pMCMC < .001) and Muslims (*b* = -0.207, HPD interval from -0.260 to -0.156, pMCMC < .001).

#### Parental Status

People with children reported less warmth toward immigrants (*b* = -0.084, HPD interval from -0.137 to -0.027, pMCMC < .001), however there was no effect of parental status on warmth toward Arabs (*b* = -0.053, HPD interval from -0.118 to 0.134, pMCMC = .122) or Muslims (*b* = -0.016, HPD interval from -0.081 to 0.053, pMCMC = .639).

#### Political Liberalism/Conservatism

Conservatives (standardized) were expected to be less warm toward immigrants (*β* = -0.133, HPD interval from -0.151 to -0.116, pMCMC < .001), Arabs (*β* = -0.193, HPD interval from -0.213 to -0.171, pMCMC < .001) and Muslims (*β* = -0.210, HPD interval from -0.232 to -0.189, pMCMC < .001). Moreover, conservatism is associated with less warmth toward both Arabs and Muslims than immigrants.

#### European Ethnic Affiliation

The data do not reveal a relationship between European ancestry and warmth toward immigrants (*b* = 0.055, HPD interval from -0.022 to 0.133, pMCMC = .166) or Arabs (*b* = -0.082, HPD interval from -0.174 to 0.005, pMCMC = .071), but European ancestry is associated with a reduction in warmth toward Muslims (*b* = -0.094, HPD interval from -0.18 to -0.002, pMCMC < .05).

#### Relationship Status

Individuals in a relationship tended to express more warmth toward immigrants (*b* = 0.075, HPD interval from 0.024 to 0.126, pMCMC < .01), but there is no evident association between relationship status and warmth toward Arabs (*b* = 0.038, HPD interval from -0.017 to 0.098, pMCMC = .193) or Muslims (*b* = 0.016, HPD interval from -0.044 to 0.075, pMCMC = .607).

#### Deprivation

Greater deprivation (standardized) predicted less warmth toward immigrants (*β* = -0.038, HPD interval from -0.059 to -0.015, pMCMC < .001), but there is no association between deprivation and warmth toward Arabs (*β* = -0.006, HPD interval from -0.032 to 0.0108 pMCMC = .673) or Muslims (*β* = -0.014, HPD interval from -0.041 to 0.011, pMCMC = .310).

#### Urban

People living in urban areas reported more warmth toward immigrants (*b* = 0.045, HPD interval from 0.000 to 0.088, pMCMC < .05) and Arabs (*b* = 0.070, HPD interval from 0.018 to 0.123, pMCMC < .01), but not Muslims (*b* = -0.048, HPD interval from -0.006 to 0.102, pMCMC = .083).

### Theoretical Variables

#### Religious Identification

Consistent with our hypotheses, religious identification (standardized) was positively associated with warmth toward Muslims (*β* = 0.084, HPD interval from 0.027 to 0.135, pMCMC < .01), and also toward Arabs (*β* = 0.140, HPD interval from 0.078 to 0.205, pMCMC < .001) and immigrants (*β* = 0.094, HPD interval from 0.030 to 0.162, pMCMC < .01).

#### Church Attendance

Frequency of church attendance (log transformed) was positively associated with warmth toward immigrants (*b* = 0.131, HPD interval from 0.084 to 0.179, pMCMC < .001), Arabs (*b* = 0.094, HPD interval from 0.040 to 0.149, pMCMC < .001) and Muslims (*b* = 0.088, HPD interval from .030 to 0.140, pMCMC < .01). Unlike some previous research [15], we found no evidence for a curvilinear relationship between frequency of religious practice and prejudice.

### Denominational Variance/Co-Variance

We report the denominational variance/covariance estimates in Tables 4 and 5. A comparison indicated that the model that included random effects for denominations was a substantially better fit than the model lacking denominational effects (*Δ* DIC = -67). Though inclusion of affiliation substantially improved model fit, consistent with Evolutionary Lag Theory, between-denomination variances were small compared to between-individual variances. To estimate within- and between-denomination variances we calculated an intra-class correlation (ICC) as the ratio of within-group variance divided by the sum of within and between-group variance:

$$\frac{\sigma_{denomination}^{2}}{\sigma_{denomination}^{2}+\;\sigma_{individual}^{2}}$$

**Table 4:** Residual Variance Structure (R-Structure units).

Units Variances	Posterior Mean	95% Lower Bounds	95% Upper Bounds
Var(Arabs)_units_	2.12	2.08	2.18
Var(Muslims)_units_	2.25	2.20	2.30
Var(Immigrant)_units_	1.54	1.51	1.58
Cov(Arabs,Muslims)_units_	1.79	1.74	1.84
Cov(Arabs, Immigrants)_units_	1.14	1.11	1.18
Cov(Muslims, Immigrants) _units_	1.22	1.19	1.26

**Table 5:** Co/Variance Solutions Denominations.

Random Intercept Denominations	Posterior Mean	95% Lower Bounds	95% Upper Bounds
Var(Arabs)_denominations_	0.027	0.002	0.063
Var(Muslims)_denominations_	0.016	0.000	0.039
Var(Immigrant)_denominations_	0.029	0.001	0.066
Cov(Arabs,Muslims)_denominations_	0.011	-0.001	0.034
Cov(Arabs,Immigrants)_denominations_	0.009	-0.010	0.035
Cov(Muslims,Immigrants)_denominations_	0.004	-0.011	0.024

ICC range from 0–1 and here indicate the amount of variance due to denominational clustering relative to total variance. Following Hadfield [103], we calculated this ratio using the posterior distributions of the intercept and religious denomination model, with denominational effects modeled as random. The ICC for Arab warmth was 0.036, the ICC for Muslim warmth was 0.031, and the ICC for immigrant warmth was 0.022. In other words, results indicate that the amount of variation in prejudice toward Arabs, Muslims and immigrants at the denominational level is 3.6%, 3.1% and 2.2% respectively. Though inclusion of demographic affiliation substantially improved model fit, between-denomination variances were generally small compared to the between-individual variances.

Consistent with Evolutionary Lag Theory, this result indicates that the expected association of religion with tolerance was on average highly robust to denominational affiliation. It is the intensity of religious commitment that predicts minority group tolerance in New Zealand, and this association applies relatively evenly across denominations. We note, however, that random intercept estimates for secular people, who for the purposes of analysis were treated as a denomination, were, as a group, somewhat higher than average for all three target groups: immigrants .142 [.024, .275], Arabs .205 [.074, .347], Muslims .141 [.017, .287].

Though religious commitment is associated with greater tolerance, and while the most strongly committed religious people are expected to have higher tolerance than demographically and ideologically matched secular people (see below), secular people are also expected to have greater tolerance than weakly committed religious people.

### Correlation of Prejudice/Acceptance Outcomes

We simultaneously modeled warmth outcomes as multivariate outcomes, which enabled us to investigate how prejudice toward the three target groups is related. The correlation between Arab and Muslim warmth was .818. The correlation between Arab and Immigrant warmth was .707. The correlation between immigrant and Muslim warmth was .656. Though Immigrant prejudice/tolerance is highly correlated with Arab and Muslim prejudice/tolerance, Arab and Muslim responses are much more strongly correlated. This suggests that prejudice/tolerance of Muslims and Arabs tends to be conflated in New Zealand.

## Interpreting the Association Between Religion and Prejudice

Using model results to compare expected outcomes for different populations is potentially misleading given that parameters are measured with error and estimated with uncertainty. Moreover, in Bayesian estimation, we infer a probabilistic range of outcomes consistent with the data, with some outcomes more probable than others. However, it may be helpful to get a sense of the relationship between religious commitment and prejudice/tolerance by comparing expected levels of warmth among demographically similar populations who differ only in their levels of religious commitment. Relatedly, we can develop a clearer intuition about the magnitude of religion’s association with prejudice by deriving predicted associations between populations of religious and secular people who differ along demographic or ideological dimensions. Here we report predicted outcomes of populations conditional on religious, demographic, and ideological status.

### Predicted Differences of Religion for Immigrant Prejudice

Among urban New Zealanders of European descent who are employed, have average levels of political conservatism, education, and deprivation, expected warmth toward immigrants for those who score at the low end of the religious identification scale (1) is 4.282. Meanwhile, expected warmth for those at the top of the religious identification scale (7) is 4.475. Among fully identified religious people who attend religious services four times per month, expected warmth toward immigrants is 4.686.

Among secular people with the highest level of education (postgraduate), but who are otherwise matched on the other baseline demographic covariates, expected warmth toward immigrants is 4.569. Among the least educated baseline secular population, expected warmth toward immigrants is 4.273. Among unemployed people, warmth toward immigrants is 4.272. Compared to baseline, expected warmth toward immigrants among the maximally conservative secular baseline population is 3.943, and expected warmth toward immigrants among maximally liberal secular people is 4.747.

### Predicted Differences of Religion for Arab Prejudice

Among urban New Zealanders of European descent who are employed, have average levels of political conservatism, education, and deprivation, expected warmth toward Arabs for those who score at the low end of the religious identification scale (1) is 3.631. Meanwhile, for those at the top of the religious identification scale (7), expected warmth toward Arabs is 3.830. Among fully identified religious people who attend religious services four times per month, expected Arab warmth is 4.105.

Among secular people with the highest level of education (postgraduate), but who are otherwise matched on the other baseline demographic covariates, expected warmth toward Arabs is 4.028. Among the least educated secular population, expected warmth toward Arabs is 3.614. Among unemployed people, expected warmth toward Arabs is 3.661. Compared to the baseline, expected warmth toward Arabs for maximally conservative secular people is 3.139, while expected warmth toward Arabs among maximally liberal people is 4.297.

### Predicted Differences of Religion for Muslim Prejudice

Among urban New Zealanders of European descent who are employed, have average levels of political conservatism, education, and deprivation, expected warmth toward Muslims for those that score at the low end of the religious identification scale (1) is 3.613. Meanwhile, for those at the top of the religious identification scale (7), expected warmth toward Muslims is 3.830. Among fully identified religious people who attend religious service four times per month, expected warmth toward Muslims is 3.971.

Among secular people with the highest level of education (postgraduate), but who are otherwise matched on the other baseline demographic covariates, expected warmth toward Muslims is 3.992. Among unemployed people, expected warmth toward Muslims is 3.535. Among the least educated baseline secular population, expected warmth toward Muslims is 3.559. Compared to baseline, expected warmth toward Muslims among maximally conservative secular people is 3.017, and expected warmth toward Muslims among maximally liberal people is 4.279.

To avoid overestimating the average differences between strongly committed religious people and secular people, we added the group level mean for “no religion” to the predicted tolerance of secular people when comparing predicted outcomes for different levels of religious commitment. Among secular people, this addition represents an increase of 0.146 (se = .001) for immigrant tolerance, an increase of 0.206 (se = .001) for Arab tolerance, and an increase of 0.145 (se = .001) for Muslim tolerance. Even with our method for conservatively estimating expected secular tolerance, we find that strong religious commitment predicts greater tolerance among people of similar demographic and ideological interests. At baseline demography and ideology, we find the expected average increase from strong religious commitment for immigrant warmth is 0.196, for Arab warmth is 0.047, and for Muslim warmth is 0.082. On average, then, strong religious commitment (defined as full religious commitment and weekly church attendance) increases expected tolerance over the secular average. However, we might examine this from another angle, that of weak religious commitment. We find that the expected average difference between secular people and weakly identified religious people who do not attend church is 0.003 for warmth toward immigrants, -0.152 for warmth toward Arabs, and is -0.135 for warmth toward Muslims. In other words, differences in tolerance between religious and secular people are driven by strongly committed religious people. Weakly committed religious people are expected to exhibit less tolerance to Arabs and Muslims, on average, than demographically matched secular people. The “something” about religion in New Zealand that unmakes prejudice would appear to be an active involvement with a religious community and identification with a tradition. The “making” of religious prejudice would appear to be a commitment to traditionalism without strong religious identification or community connection.

Taken collectively, results suggest that in New Zealand, high levels of religious identification and church attendance are associated with substantial reductions in minority-group prejudice, and that such tolerance extends to members of a poorly tolerated religious minority group, Muslims.

## Discussion

We opened with Allport’s claim that there is something about religion that makes prejudice and something that unmakes prejudice [1]. We noticed that previous attempts to explain variation in the association between religion and prejudice have proved inconclusive. We proposed an Evolutionary Lag Theory, which holds that historical geo-political conflict and peace are better predictors of religious tolerance and prejudice than denominational affiliation. The theory also predicts that in peaceable regions, strong religious commitment will tend to unify people across ethnic and religious boundaries rather than have the reverse or null effect. Here we investigated the relationship between the religious commitments of non-Muslims and levels of anti-Muslim prejudice in New Zealand. New Zealand is an ideal setting in which to investigate religion and Muslim tolerance: New Zealand is incredibly diverse, socially progressive and tolerant, has a history of peace, a history of inter-faith institutions, and a large secular population.

Our study addresses three methodological gaps. First, multi-level models that address denominational clustering are uncommon, yet un-modeled dependencies arising from participants’ affiliation with religious traditions violate the independency assumption of regression and ANOVA [100]. By modeling denominational membership as a random effect we are able to both estimate and adjust for clustering arising from denominational affiliation. Second, rich and informative demographic covariates have largely been missing from statistical models. We argue that research that downplays demographic factors risks imparting an incomplete account, because the relative contribution of religion is not assessed against a background of other factors associated with prejudice. The absence of demographic information may also result in misleading results by artificially inflating, deflating, or even reversing the sign of coefficients meant to assess religion. By including key demographic covariates, the results here adjust for variation in responses explained by factors that previous research has shown to be associated with prejudice, and give greater confidence about the sign and magnitude of religion covariates. Furthermore, by inclusion of demographic covariates, we are able to compare the magnitude of religion’s association with known drivers of prejudice and tolerance. Third, previous research lacked appropriate within-individual comparisons between anti-Muslim prejudice and other types of prejudice. We argue that simultaneously modeling associations between demographic and religion covariates (predictors) on anti-Muslim prejudice and appropriate comparison groups (multivariate outcomes) affords more informative models about the mechanisms that influence prejudice.

In support of Evolutionary Lag Theory, we found that strong religious faith among non-Muslims is associated with greater tolerance of Muslims. Indeed, the expected increase in tolerance from strong religious commitment is similar to that expected from obtaining a postgraduate education. Additionally, we find that adjusting for denominations substantially improved model fit. This suggests that there are benefits from including denominational affiliation as a random effect. However, we also found that the magnitude of between-denominational variation in our sample is relatively small across the three prejudice targets. Consistent with Evolutionary Lag Theory, this finding suggests that the association of strong religious commitment with tolerance tends to be quite general in a peaceful society such as New Zealand, and is highly robust to denominational differences.

Similarly, we found that religious commitments are associated with increased tolerance toward all three target groups examined. That religious based tolerance extends to ethnic out-groups (Arabs and Immigrants) suggests that our findings are not due to the existence of an Abrahamic Religions in-group. Consistent with Evolutionary Lag Theory, high levels of commitment with a religious group were found to be associated with out-group tolerance, rather than out-group prejudice. While these findings are consistent with predictions derived from our theoretical model, future cross-cultural studies are necessary for improved evaluation.

Finally, we also found that anti-Arab and anti-Muslim sentiments are strongly correlated, and both targets exhibit much lower average tolerance than does the immigrant target, implying the category “Muslim” and “Arab” may tend to be conflated in the minds of non-Muslims. This result is at striking odds with the known cultural and ethnic diversity of Muslims in this country.

### Theoretical Implications

We began noting that Clash of Civilizations models usefully point to historical path dependencies in Muslim-Western conflicts, but such theories fail to explain regional variation, particularly in peaceful societies with little history of violent conflict. Religious Orientations theories attempts to explain individual variation, but similar to Clash of Civilizations theories, fail to explain cross-cultural variation. It is not enough to claim that social conflict is complex and the result of multiple processes, including history, economics and politics [105]. We argue that evolutionary theory offers a framework for understanding how historical and psychological factors combine to influence conflict and peace.

Clash of Civilizations theories, as well as the model we advance here, are in many ways similar to prominent psychological theories that attempt to explain why humans are sometimes prejudiced toward the members of other groups, such as social identity theory [106] or social dominance theory [107]. For example, these theories expect that several key environmental variables such as group size, social inequality, and perceived threats all influence the relationship between in-group identification and out-group prejudice. Evolutionary Lag Theory also expects these variables to influence prejudice, as they are key indicators of threats to collective resources. We suggest that evolutionary theory may contribute to the psychology of prejudice by clarifying how the strategic dimensions of prejudice respond to environmental contingencies. Moreover, evolutionary theory helps to refine empirical predictions and to make sense of ambiguous findings in previous research. Here we use evolutionary theory to reveal a potentially useful resource for mending intergroup conflict in peaceable settings, whose role remains ambiguous in the social psychology of prejudice, namely religion. That the religious commitments of non-Muslims may be channeled to increase tolerance to Muslims holds practical importance in Western democracies that are struggling with anti-Muslim sentiments.

### Practical Implications

A core motivation for this study is to understand how prejudice arises in peaceful democracies in the absence of personal experiences of violent conflict. The absence of violence or open clashes should not be construed as evidence that prejudice is absent and that minority groups are not being harmed. Nor should the absence of violent conflict be taken as an indicator that future violence is unlikely. Despite the peaceable and affluent setting in New Zealand, we find that anti-Muslim sentiments are widespread. Orthogonal literatures reveal that experiencing discrimination is associated with a range of negative outcomes, such as poorer health, increased mortality [108], and experiences of unfair treatment in the criminal justice system [109].

By assessing multivariate prejudice outcomes we obtained comparative inference for the relative strength of anti-Muslim prejudice compared to other types of minority-group prejudice within individuals. This comparative assessment of prejudice showed a conflation of Arabs and Muslims. That both Arabs and Muslims are the recipients of less warmth than immigrants suggests that both groups are the recipients of intolerance. This conflation should be surprising given that the overwhelming majority of New Zealand’s Muslims were not born in the Middle East or North Africa (76.7%) [56], and only 19.8% of the world’s Muslims are from the Middle East or North Africa [110]. Similar responses to Arabs and Muslims are thus unlikely to result from previous experiences and reflect a lack of knowledge about Islam. It is likely that many New Zealanders automatically assume that Arabs are Muslims, and vice versa, though the overlap between these groups is limited. Our study identifies a basic ethno-religious conflation, and suggests that investigation into the source of these conflations is imperative.

It might be easy to overlook religion as a resource for Muslim tolerance because the history of Muslim/Arab-Christian/Western relations often focuses on violent encounter. Consistent with our theoretical predictions, we observed a positive association between religious identification and warmth toward Muslims. We emphasize that Evolutionary Lag Theory does not predict that religious commitment will always and invariably increase tolerance. Nor does it predict that denominational differences will always and invariably turn out to be negligible. Quite the opposite. In conflict-prone religions with a history of religious conflict, Evolutionary Lag Theory predicts that strong religious commitment will tend to inhibit social acceptance, intensify social divisions, exacerbate conflict, and provide symbolic markers that accentuate group boundaries, as recent studies observe [20]. However, the present study is important because it reveals how religion may be a resource for social unification in peaceable regions. Despite religion’s association with violence in violent regions, we find that strong religious commitments in peaceful societies may be an engine for increasing social acceptance, including the acceptance of Muslims.

## Supporting Information

S1 Fig A comparison of MCMC models run on three datasets. (TIF)

S1 File Pairwise Deleted Dataset Results. (DOCX)

S2 File Amelia Imputed Dataset Results. (DOCX)

S1 Table Predictors of Tolerance for Immigrants, Arabs and Muslims (pairwise deleted data). (DOCX)

S2 Table Residual Variance Structure (R-Structure units). (DOCX)

S3 Table Co/Variance Solutions Denominations. (DOCX)

S4 Table Predictors of Tolerance for Immigrants, Arabs and Muslims (AMELIA Imputed data). (DOCX)

S5 Table Number of Individuals Affiliated with each Denomination. (DOCX)
