# Supplementary figures and images for: Religion and the Unmaking of Prejudice toward Muslims: Evidence from a Large National Sample
Source: PLoS One. 2016 Mar 9;11(3):e0150209. doi: 10.1371/journal.pone.0150209 (PMC4784898; doi:10.1371/journal.pone.0150209)

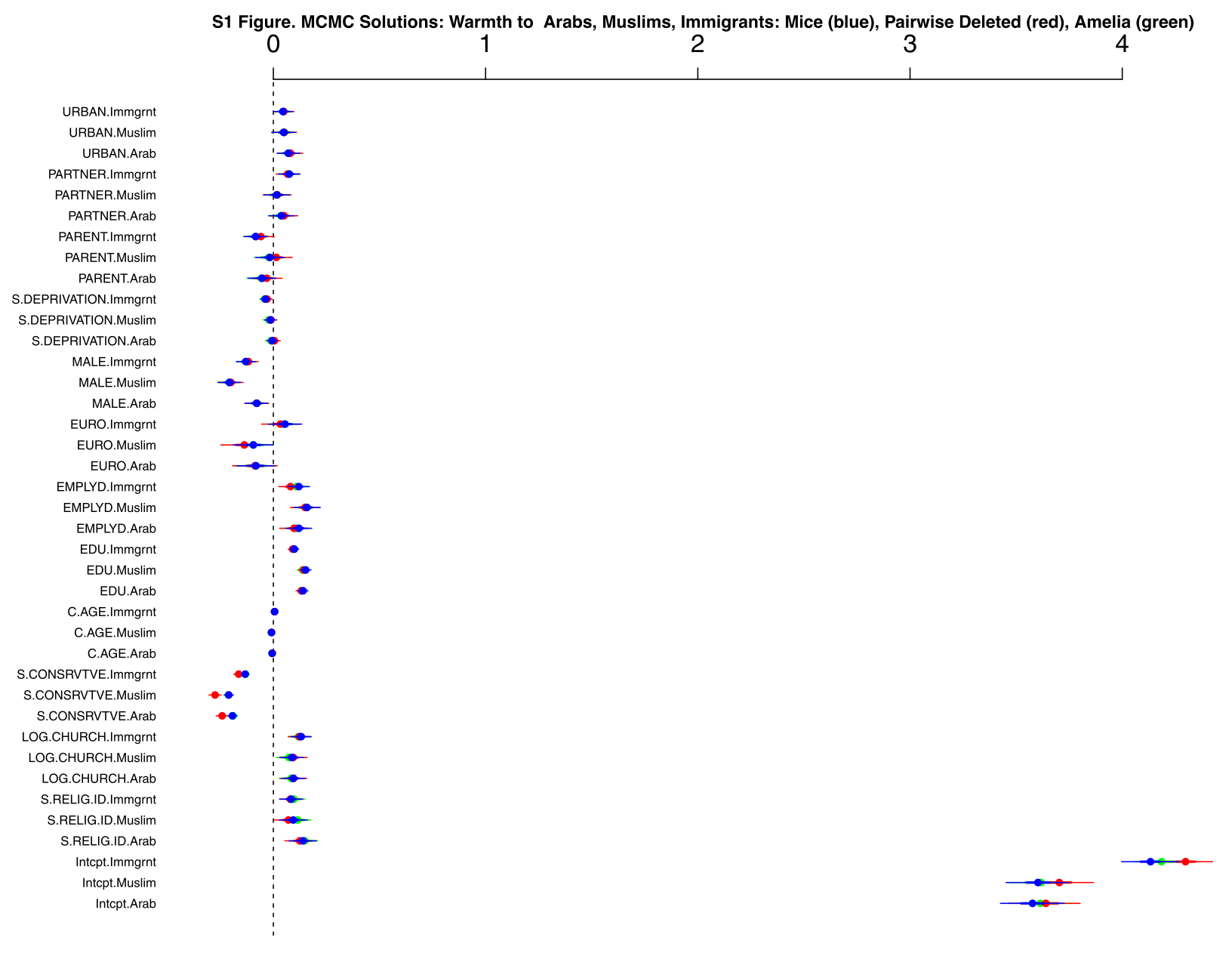

Supplement: S1 Fig — (TIF) [file pone.0150209.s001.tif]
