# Supplementary material for: Religion and the Unmaking of Prejudice toward Muslims: Evidence from a Large National Sample
Source: PLoS One. 2016 Mar 9;11(3):e0150209. doi: 10.1371/journal.pone.0150209 (PMC4784898; doi:10.1371/journal.pone.0150209)
Supplement: S1 File — (DOCX) [file pone.0150209.s002.docx]

**S1 File. Pairwise Deleted Dataset Results**

The results of a multivariate regression model predicting warmth toward immigrants, Arabs and Muslims using the pairwise deleted dataset (*N* = 11,405) are presented in S1 Table. The location effects for the intercepts were: warmth toward Muslims *b* = 3.703, [HPD interval from 3.541 to 3.864], Arabs *b* = 3.639, HPD interval from [3.471 to 3.805], and immigrants *b* = 4.298, [HPD interval from 4.167 to 4.428]. We report the average denominational variance/covariances in S2 Table and S3 Table.

In general, models run on the pairwise data make little difference for inference relative to models run on the imputed dataset. There was one difference, however: 1) with the imputed dataset there is some evidence for a relationship between parental status and decreased warmth toward immigrants (*b* = -0.084, HPD interval from -0.137 to -0.027, pMCMC < .001), though with the pairwise deleted dataset the evidence for this association is weaker (*b* = -0.059, HPD interval from -0.122 to 0.004, pMCMC = .065). Full results of the model run on the pairwise deleted dataset are described below.

**Demographic Indicators**

**Age**

Each year of age was associated with slightly more warmth toward immigrants (*b* = 0.006, HPD interval from 0.004 to 0.008, pMCMC < .001), but less warmth toward Arabs (*b* = -0.005, HPD interval from -0.008 to -0.003, pMCMC < .001) and Muslims (*b* = -0.008, HPD interval from -0.011 to -0.006, pMCMC < .001).

**Education**

Educated people were warmer toward immigrants (*b* = 0.091, HPD interval from 0.072 to 0.113, pMCMC < .001), Arabs (*b* = 0.132, HPD interval from 0.106 to 0.158, pMCMC < .001) and Muslims (*b* = 0.143, HPD interval from 0.117 to 0.168, pMCMC < .001).

**Employment**

Employment was associated with more warmth toward Muslims (*b* = 0.149, HPD interval from 0.082 to 0.217, pMCMC < .001), Arabs (*b* = 0.098, HPD interval from 0.029 to 0.163, pMCMC < .01) and immigrants (*b* = 0.081, HPD interval from 0.025 to 0.134, pMCMC < .01).

**Gender**

Men reported less warmth toward immigrants (*b* = -0.118, HPD interval from -0.164 to -0.073, pMCMC < .01), Arabs (*b* = -0.078, HPD interval from -0.137 to -0.024, pMCMC < .001) and Muslims (*b* = -0.200, HPD interval from -0.258 to -0.142, pMCMC < .001).

**Parental Status**

Parental status was not associated with warmth toward immigrants (*b* = -0.059, HPD interval from -0.122 to 0.004, pMCMC = .065), Arabs (*b* = -0.031, HPD interval from -0.105 to 0.040, pMCMC = .396) or Muslims (*b* = 0.014, HPD interval from -0.063 to 0.085, pMCMC = .722).

**Political Liberalism/Conservatism**

Conservatives (standardized) were expected to be less warm toward immigrants (*β* = -0.164, HPD interval from -0.188 to -0.140, pMCMC < .001), Arabs (*β* = -0.241, HPD interval from -0.269 to -0.213, pMCMC < .001) and Muslims (*β* = -0.275, HPD interval from -0.302 to -0.244, pMCMC < .001).

**European Ethnic Affiliation**

People of European ethnic affiliation reported less warmth toward Muslims (*b* = -0.137, HPD interval from -0.246 to -0.027, pMCMC < .05). There was no relationship between European ancestry and warmth toward immigrants (*b* = 0.033, HPD interval from -0.058 to 0.117, pMCMC = .451) or Arabs (*b* = -0.087, HPD interval from -0.193 to 0.016, pMCMC = .108).

**Relationship Status**

People in a relationship tended to express more warmth toward immigrants (*b* = 0.067, HPD interval from 0.015 to 0.124, pMCMC < .05), but not Muslims (*b* = 0.018, HPD interval from -0.048 to 0.084, pMCMC = .590) or Arabs (*b* = 0.050, HPD interval from -0.0164to 0.115, pMCMC = .131).

**Deprivation/Socio-Economic Status**

Increasing deprivation (standardized) predicted less warmth toward immigrants (*β* = -0.029, HPD interval from -0.053 to -0.006, pMCMC < .05), but not Muslims (*β* = -0.011, HPD interval from -0.040 to 0.017, pMCMC = .444) or Arabs (*β* = 0.004, HPD interval from -0.024 to 0.032, pMCMC = .713).

**Urban**

People living in urban areas reported more warmth toward Arabs (*b* = 0.080, HPD interval from 0.025 to 0.141, pMCMC < .001), but not immigrants (*b* = 0.049, HPD interval from 0.001 to 0.099, pMCMC = .051) or Muslims (*b* = 0.050, HPD interval from -0.007 to 0.111, pMCMC = .102).

**Theoretical Variables**

**Religious Identification**

Consistent with our hypotheses, religious identification (standardized) was positively associated with warmth toward Muslims (*β* = 0.077, HPD interval from 0.001 to 0.142, pMCMC < .05), and also toward Arabs (*β* = 0.124, HPD interval from 0.052 to 0.195, pMCMC < .001) and immigrants (*β* = 0.080, HPD interval from 0.027 to 0.132, pMCMC < .05).

**Church Attendance**

Frequency of church attendance (log transformed) was also positively associated with warmth toward immigrants (*b* = 0.122, HPD interval from 0.0872 to 0.177, pMCMC < .001), Arabs (*b* = 0.096, HPD interval from 0.033 to 0.157, pMCMC < .01) and Muslims (*b* = 0.094, HPD interval from .030 to 0.157, pMCMC < .01).
