# Supplementary material for: Religion and the Unmaking of Prejudice toward Muslims: Evidence from a Large National Sample
Source: PLoS One. 2016 Mar 9;11(3):e0150209. doi: 10.1371/journal.pone.0150209 (PMC4784898; doi:10.1371/journal.pone.0150209)
Supplement: S2 File — (DOCX) [file pone.0150209.s003.docx]

S2 File. Amelia Imputed Dataset Results

The results of a multivariate regression model predicting warmth toward immigrants, Arabs and Muslims using the Amelia package to impute the dataset are presented in S4 Table. The location effects for the intercepts were: warmth toward Muslims *b* = 3.617, [HPD interval from 3.476 to 3.759], Arabs *b* = 3.614, [HPD interval from 3.467 to 3.763], and immigrants *b* = 4.184, [HPD interval from 4.061 to 4.312].

In general, models run on data imputed with the Mice and Amelia packages return the same results. Full results of the model run on the Amelia imputed dataset are described below.

**Demographic Indicators**

**Age**

Each year of age was associated with slightly more warmth toward immigrants (b = 0.006, HPD interval from 0.004 to 0.007) but less warmth toward Arabs (b = -0.006, HPD interval from -0.008 to -0.004) and Muslims (b = -0.009, HPD interval from -0.011 to -0.007).

**Education**

Educated people were warmer toward immigrants (b = 0.096, HPD interval from 0.077 to 0.116), Arabs (b = 0.134, HPD interval from 0.111 to 0.157) and Muslims (b = 0.138, HPD interval from 0.114 to 0.161).

**Employment**

Employment was associated with more warmth toward Muslims (b = 0.153, HPD interval from 0.1092to 0.215), Arabs (b = 0.110, HPD interval from 0.050 to 0.171) and immigrants (b = 0.109, HPD interval from 0.058 to 0.161).

**Gender**

Men reported less warmth toward immigrants (b = -0.127, HPD interval from -0.170 to -0.082), Arabs (b = -0.078, HPD interval from -0.130 to -0.027) and Muslims (b = -0.207, HPD interval from -0.262 to -0.151). Moreover, men reported less warmth toward Muslims than Arabs and immigrants.

**Parental Status**

Parents reported less warmth toward immigrants (b = -0.082, HPD interval from -0.140 to -0.024). There is no association between parental status and warmth toward Arabs (b = -0.058, HPD interval from -0.123 to 0.009) or Muslims (b = -0.021, HPD interval from -0.089 to 0.049).

**Political Liberalism/Conservatism**

Conservatives (standardized) were expected to be less warm toward immigrants (*β* = -0.131, HPD interval from -0.149 to -0.113), Arabs (*β* = -0.190, HPD interval from -0.210 to -0.168) and Muslims (*β* = -0.212, HPD interval from -0.233 to -0.190). Moreover, conservatism is associated with less warmth toward both Arabs and Muslims than Immigrants.

**European Ethnic Affiliation**

There is no association between European ethnic affiliation and warmth toward immigrants (b = 0.051, HPD interval from -0.039 to 0.130) or Arabs (b = -0.083, HPD interval from -0.174 and 0.007), but those of European descent reported less warmth toward Muslims (b = -0.095, HPD interval from -0.188 to -0.003).

**Relationship Status**

People in a relationship tended to express more warmth toward immigrants than single people (b = 0.072, HPD interval from 0.023 to 0.125), but there is no association between relationship status and warmth toward Arabs (b = 0.038, HPD interval from -0.023 to 0.097) or Muslims (b = 0.016, HPD interval from -0.047 to 0.078).

**Deprivation/Socio-Economic Status**

Increasing deprivation (standardized) predicted less warmth toward immigrants (*β* = -0.041, HPD interval from -0.064 to -0.019), but there is no evident association between deprivation and warmth toward Muslims (*β* = -0.021, HPD interval from -0.049 to 0.006) or Arabs (*β* = -0.012, HPD interval from -0.037 to 0.014).

**Urban**

People living in urban areas reported more warmth toward immigrants (b = 0.048, HPD interval from 0.003 to 0.093) and Arabs (b = 0.072, HPD interval from 0.019 to 0.124), but there is no evidence for a relationship between urban dwelling and warmth toward Muslims (b = 0.053, HPD interval from -0.001 to 0.107).

**Theoretical Variables**

**Religious Identification**

Consistent with our hypotheses, religious identification (standardized) was positively associated with warmth toward Muslims (*β* = 0.115, HPD interval from 0.053 to 0.174), and also toward Arabs (*β* = 0.148, HPD interval from 0.088 to 0.208) and immigrants (*β* = 0.097, HPD interval from 0.046 to 0.149).

**Church Attendance**

Frequency of church attendance (log transformed) was also positively associated with warmth toward immigrants (b = 0.119, HPD interval from 0.071 to 0.166), Arabs (b = 0.083, HPD interval from 0.028 to 0.140) and Muslims (b = 0.072, HPD interval from .015 to 0.131).
