# Supplementary material for: Religion and the Unmaking of Prejudice toward Muslims: Evidence from a Large National Sample
Source: PLoS One. 2016 Mar 9;11(3):e0150209. doi: 10.1371/journal.pone.0150209 (PMC4784898; doi:10.1371/journal.pone.0150209)
Supplement: S1 Table — (DOCX) [file pone.0150209.s004.docx]

S1 Table. Predictors of Tolerance for Immigrants, Arabs and Muslims (pairwise deleted data).

|  | **_Warmth toward Immigrants_** | | | | **_Warmth toward Arabs_** | | | | **_Warmth toward Muslims_** | | | |
| --- | --- | --- | --- | --- | --- | --- | --- | --- | --- | --- | --- | --- |
|  | **Posterior Mean** | **95 % Lower Bounds** | **95 % Upper Bounds** | **pMCMC** | **Posterior Mean** | **95 % Lower Bounds** | **95 % Upper Bounds** | **pMCMC** | **Posterior Mean** | **95 % Lower Bounds** | **95 % Upper Bounds** | **pMCMC** |
| **Intercept** | 4.298 | 4.167 | 4.428 | *** | 3.639 | 3.471 | 3.805 | *** | 3.703 | 3.541 | 3.864 | *** |
| **Age (centered)** | 0.006 | 0.004 | 0.008 | *** | -0.005 | -0.008 | -0.003 | *** | -0.008 | -0.011 | -0.006 | *** |
| **Education** | 0.091 | 0.072 | 0.113 | *** | 0.132 | 0.106 | 0.158 | *** | 0.143 | 0.117 | 0.168 | *** |
| **Employed** | 0.081 | 0.025 | 0.134 | ** | 0.098 | 0.029 | 0.163 | ** | 0.149 | 0.082 | 0.217 | *** |
| **Gender** | -0.118 | -0.164 | -0.073 | *** | -0.078 | -0.137 | -0.024 | ** | -0.200 | -0.258 | -0.142 | *** |
| **Parental Status** | -0.059 | -0.122 | 0.004 |  | -0.031 | -0.105 | 0.040 |  | 0.014 | -0.063 | 0.085 |  |
| **Political Conservatism (standardized)** | -0.164 | -0.188 | -0.140 | *** | -0.241 | -0.269 | -0.213 | *** | -0.275 | -0.302 | -0.244 | *** |
| **European** | 0.033 | -0.058 | 0.117 |  | -0.087 | -0.193 | 0.016 |  | -0.137 | -0.246 | -0.027 | * |
| **Partner** | 0.067 | 0.015 | 0.124 | * | 0.050 | -0.014 | 0.115 |  | 0.018 | -0.048 | 0.084 |  |
| **Deprivation (standardized)** | -0.029 | -0.053 | -0.006 | * | 0.005 | -0.024 | 0.032 |  | -0.011 | -0.040 | 0.017 |  |
| **Urban** | 0.049 | 0.001 | 0.099 |  | 0.080 | 0.025 | 0.141 | ** | 0.050 | -0.007 | 0.111 |  |
| **Religious ID (standardized)** | 0.080 | 0.027 | 0.132 | ** | 0.124 | 0.052 | 0.195 | *** | 0.071 | 0.001 | 0.142 | * |
| **Church Attendance (log)** | 0.122 | 0.072 | 0.177 | *** | 0.096 | 0.033 | 0.157 | ** | 0.094 | 0.030 | 0.157 | ** |

S1 Table Key: * = pMCMC < 0.05; ** = pMCMC < .01; *** = pMCMC < .001
