# Supplementary material for: Religion and the Unmaking of Prejudice toward Muslims: Evidence from a Large National Sample
Source: PLoS One. 2016 Mar 9;11(3):e0150209. doi: 10.1371/journal.pone.0150209 (PMC4784898; doi:10.1371/journal.pone.0150209)
Supplement: S2 Table — (DOCX) [file pone.0150209.s005.docx]

S2 Table. Residual Variance Structure (R-Structure units).

| **Units Variances** | **Posterior Mean** | **95 % Lower Bounds** | **95 % Upper Bounds** |
| --- | --- | --- | --- |
| Var(Arabs)_units_ | 2.031 | 1.977 | 2.084 |
| Var(Muslims)_units_ | 2.162 | 2.106 | 2.217 |
| Var(Immigrant)_units_ | 1.435 | 1.396 | 1.472 |
| Cov(Arabs,Muslims)_units_ | 1.720 | 1.668 | 1.768 |
| Cov(Arabs,Immigrants)_units_ | 1.078 | 1.040 | 1.115 |
| Cov(Muslims,Immigrants) _units_ | 1.159 | 1.120 | 1.198 |
