# Supplementary material for: Religion and the Unmaking of Prejudice toward Muslims: Evidence from a Large National Sample
Source: PLoS One. 2016 Mar 9;11(3):e0150209. doi: 10.1371/journal.pone.0150209 (PMC4784898; doi:10.1371/journal.pone.0150209)
Supplement: S3 Table — (DOCX) [file pone.0150209.s006.docx]

**S3 Table. Co/Variance Solutions Denominations.**

|  | **Posterior Mean** | **95 % Lower Bounds** | **95 % Upper Bounds** |
| --- | --- | --- | --- |
| Var(Arabs)_denominations_ | 0.027 | 0.002 | 0.073 |
| Var(Muslims)_denominations_ | 0.010 | 0.000 | 0.028 |
| Var(Immigrant)_denominations_ | 0.006 | 0.000 | 0.016 |
| Cov(Arabs,Muslims)_denominations_ | 0.008 | -0.005 | 0.029 |
| Cov(Arabs,Immigrants)_denominations_ | 0.005 | -0.005 | 0.020 |
| Cov(Muslims,Immigrants)_denominations_ | 0.004 | -0.003 | 0.013 |
