# Supplementary material for: Religion and the Unmaking of Prejudice toward Muslims: Evidence from a Large National Sample
Source: PLoS One. 2016 Mar 9;11(3):e0150209. doi: 10.1371/journal.pone.0150209 (PMC4784898; doi:10.1371/journal.pone.0150209)
Supplement: S4 Table — (DOCX) [file pone.0150209.s007.docx]

**S4 Table. Predictors of Tolerance for Immigrants, Arabs and Muslims (AMELIA imputed data).**

|  | **_Warmth toward Immigrants_** | | | **_Warmth toward Arabs_** | | | **_Warmth toward Muslims_** | | |
| --- | --- | --- | --- | --- | --- | --- | --- | --- | --- |
|  | Posterior Mean | 95 % Lower Bounds | 95 % Upper Bounds | Posterior Mean | 95 % Lower Bounds | 95 % Upper Bounds | Posterior Mean | 95 % Lower Bounds | 95 % Upper Bounds |
| **Intercept** | 4.184 | 4.061 | 4.312 | 3.614 | 3.467 | 3.763 | 3.617 | 3.476 | 3.759 |
| **Age (centered)** | 0.006 | 0.004 | 0.007 | -0.006 | -0.008 | -0.004 | -0.009 | -0.011 | -0.007 |
| **Education** | 0.096 | 0.077 | 0.116 | 0.134 | 0.111 | 0.157 | 0.138 | 0.114 | 0.161 |
| **Employed** | 0.109 | 0.058 | 0.161 | 0.110 | 0.050 | 0.171 | 0.153 | 0.092 | 0.215 |
| **Gender** | -0.127 | -0.170 | -0.082 | -0.078 | -0.130 | -0.027 | -0.207 | -0.262 | -0.151 |
| **Parental Status** | -0.082 | -0.140 | -0.024 | -0.058 | -0.123 | 0.009 | -0.021 | -0.089 | 0.049 |
| **Political Conservatism (standardized)** | -0.131 | -0.149 | -0.113 | -0.190 | -0.210 | -0.168 | -0.212 | -0.233 | -0.190 |
| **European** | 0.051 | -0.029 | 0.130 | -0.083 | -0.174 | 0.007 | -0.095 | -0.188 | -0.003 |
| **Partner** | 0.072 | 0.023 | 0.125 | 0.038 | -0.023 | 0.097 | 0.016 | -0.047 | 0.078 |
| **Deprivation (standardized)** | -0.041 | -0.064 | -0.019 | -0.012 | -0.037 | 0.014 | -0.021 | -0.049 | 0.006 |
| **Urban** | 0.048 | 0.003 | 0.093 | 0.072 | 0.019 | 0.124 | 0.053 | -0.001 | 0.107 |
| **Religious ID (standardized)** | 0.097 | 0.046 | 0.149 | 0.148 | 0.088 | 0.208 | 0.115 | 0.053 | 0.174 |
| **Church Attendance (log)** | 0.119 | 0.071 | 0.166 | 0.083 | 0.028 | 0.140 | 0.072 | 0.015 | 0.131 |
