# Supplementary material for: Religion and the Unmaking of Prejudice toward Muslims: Evidence from a Large National Sample
Source: PLoS One. 2016 Mar 9;11(3):e0150209. doi: 10.1371/journal.pone.0150209 (PMC4784898; doi:10.1371/journal.pone.0150209)
Supplement: S5 Table — (DOCX) [file pone.0150209.s008.docx]

S5. Table. Number of Individuals Affiliated with each Denomination

| Denomination | *n* |
| --- | --- |
| Anglican | 899 |
| Apostolic Church of New Zealand | 2 |
| Assemblies of God | 11 |
| Baha’i | 13 |
| Baptist NEC | 3 |
| Baptist NFD | 97 |
| Born Again | 10 |
| Brethren NEC | 1 |
| Brethren NFD | 4 |
| Buddhist NEC | 5 |
| Buddhist NFD | 93 |
| Catholic NEC | 23 |
| Catholic NFD | 906 |
| Christadelphian | 2 |
| Christian NEC | 30 |
| Christian NFD | 1492 |
| Christian Outreach | 1 |
| Christian Science | 2 |
| Church Of Christ NFD | 1 |
| Congregational | 3 |
| Elim | 5 |
| Evangelical | 6 |
| Greek Orthodox | 4 |
| Hare_Krishna | 1 |
| Hindu NEC | 4 |
| Hindu_NFD | 13 |
| Jehovah’s Witnesses | 52 |
| Judaism Jewish | 12 |
| Latter Day Saints | 126 |
| Lutheran | 6 |
| Mahikari | 3 |
| Maori Christian NEC | 13 |
| Maori Christian NFD | 5 |
| Maori Religion NEC | 5 |
| Maori Religion NFD | 10 |
| Methodist NEC | 2 |
| Methodist NFD | 69 |
| Missing Data | 374 |
| Nature and Earth Based Religions NEC | 21 |
| Nature and Earth Based Religions NFD | 3 |
| New Age NFD | 6 |
| New Life Centres | 6 |
| Object to answering | 2 |
| Open Brethren | 11 |
| Other New Age Religions NEC | 10 |
| Other Religions NEC | 8 |
| Pantheist | 1 |
| Pentecostal NEC | 5 |
| Pentecostal NFD | 36 |
| Plymouth Brethren | 5 |
| Presbyterian | 349 |
| Protestant NFD | 9 |
| Rastafarianism | 2 |
| Ratana | 86 |
| Reformed | 6 |
| Religion Unidentifiable | 41 |
| Religious Society of Friends Quaker | 14 |
| Response Outside Scope | 40 |
| Revival Centres | 2 |
| Ringatu | 20 |
| Roman Catholic | 256 |
| Russian Orthodox | 1 |
| Salvation Army | 34 |
| Seventh Day Adventist | 27 |
| Sikh | 3 |
| Spiritualist | 48 |
| Taoism | 6 |
| Tongan Methodist | 1 |
| Unitarian | 1 |
| Uniting Union Church | 10 |
| Vineyard Christian Fellowship | 4 |
| Wiccan | 4 |
| Yoga | 8 |
| Zen Buddhist | 5 |

S5 Table Key: NEC, not elsewhere classified; NFD, not further defined.
